# Stroke, Dementia, and Atrial Fibrillation: From Pathophysiologic Association to Pharmacological Implications

**DOI:** 10.3390/medicina56050227

**Published:** 2020-05-10

**Authors:** Vincenzo Russo, Riccardo Vio, Riccardo Proietti

**Affiliations:** 1 Cardiology Unit, Department of Medical Translational Sciences, University of Campania “Luigi Vanvitelli”—Monaldi Hospital, Via Leonardo Bianchi, 80131 Naples, Italy; 2 Department of Cardiac, Thoracic, and Vascular Sciences, University of Padua, Via Giustiniani 2, 35121 Padua, Italy; riccardo.vio.1@gmail.com (R.V.); riccardo.proieti@unidp.it (R.P.)

The impact of stroke and dementia on disability and death is a major contemporary health issue. The proportion of ischemic strokes related to atrial fibrillation (AF) ranges from one-sixth to one-third, with the highest percentage reported among octogenarians [1]. Since AF episodes may be asymptomatic and misdiagnosed, patients at increased risk of AF should be screened for the early detection of the silent AF, in order to avoid preventable cardioembolic strokes. Furthermore, both overt and silent ischemic strokes occurring in AF patients may cause vascular dementia, the more prevalent subset of dementia in this population, impacting on cognitive function [2]. 

Lastly, the risk of cerebrovascular events in AF patients is unrelated to the burden or persisting of arrhythmia [3]; which implies that both paroxysmal and permanent or persistent AF share the same risk of stroke and deserve anticoagulation therapy according to the patient’s CHA2DS2VASc risk score.

Until 2011, vitamin K antagonists (VKAs) represented the standard anticoagulant therapy for reducing thromboembolic risk in AF patients. However, the patient compliance to VKA treatment in real-world setting is undermined by their slow onset of action, variable pharmacologic effects, several food and drug interactions. Moreover, VKA therapy requires serial target international normalized ratio (INR) monitoring to optimize its clinical management [4,5].

To overcome these issues, non-vitamin K oral antagonists (NOACs) have been developed and are now preferred over VKA therapy in AF patients at increased risk of stroke, excluding mechanical heart valve recipients, and patients with moderate to severe rheumatic mitral stenosis [6].

NOACs have replaced VKAs therapy in several clinical settings based on phase III randomized clinical trial (RCT) results [7], and on real-world data, including AF patients with clinical features excluded from RCTs [8,9,10,11].

Moreover, NOACs are an effective and safe alternative to the best possible conventional treatment with VKAs among AF patients undergoing direct current cardioversion or percutaneous coronary interventions [12,13,14].

The prevalence of AF increases with advancing age, together with ischemic and hemorrhagic stroke occurrence. AF is associated with either vascular or non-vascular dementia [15], particularly among octogenarians causing their exclusion from clinical trials for doubts in adherence to treatment.

However, available data support the concept that very elderly AF patients may benefit from the increased effectiveness and safety of NOACs, likewise for the general population [16,17].

In this issue of *Medicina*, several authors gathered together the most recent evidence on the association between cerebrovascular events, cognitive impairment, and AF. Al Turki et al. produced an elegant review on the topic of subclinical AF, as detected by cardiac devices in asymptomatic patients [18]. Dual-chambered devices have the potential to identify so-called atrial high-rate episodes (AHRE), which have been repeatedly linked to an augmented risk of stroke. The impact of duration and burden of AHRE on the risk of stroke, as well as the tendency to progress to longer episodes, are described in the review. The management of such episodes remains a matter of debate, but promising ongoing trials will soon unveil the kind of AHRE that require anticoagulation therapy according to a patient’s risk profile. 

Gallinoro et al. explored recent reports suggesting that AF may predict cognitive impairment and dementia, even in stroke-free patients [19]. The comprehension of the underpinning mechanisms could provide an insight into future therapeutic targets. Cerebral hypoperfusion is just one of the aspects that links AF and cognitive decline, but as far as it depends on the perpetuation of arrhythmia, the authors suggest the potential usefulness of restoring and maintaining sinus rhythm.

Poggesi et al. present the design, methodology, and preliminary results of the Strat-AF study [20]. This prospective observational study is primarily aimed at investigating how circulating biomarkers might help to further stratify the cerebral bleeding risk of AF patients on oral anticoagulation therapy. Apart from the primary endpoint, secondary outcomes include either ischemic or non-ischemic stroke occurrence and functional, cognitive, and motor status; the Strat-AF study aspires to ameliorate the available stroke prediction models by fostering the inclusion of several biomarkers.

Another important contribution by Al Turki et al. illustrates how, and in which measure, cardiovascular comorbidities may be related to AF and cognitive decline, with particular reference to metabolic disorders including diabetes mellitus and obesity [21]. The authors highlight the importance of the atrial cardiomyopathy driven by metabolic syndromes. According to this perspective, such fibrotic changes in the atria, together with chamber dilation, leads to the onset of AF and subsequently to cerebrovascular thromboembolic events. Nonetheless, cardiovascular and metabolic comorbidities prompt cognitive impairment and dementia, other than vascular, through different pathways not yet fully understood. There is mounting evidence that the antihyperglycemic therapy used for the treatment of diabetes mellitus can alter the occurrence of stroke and AF. This aspect has been extensively reviewed by Lăcătuşu et al. [22], who pointed out paradoxical effects for different antidiabetic drugs, calling for new trials aimed to deepen our understanding in the field. 

In conclusion, the main scope of the present Special Issue is to summarize the most updated evidence regarding the interplay between AF, cognitive impairment, and cerebrovascular events. Given the social impact of stroke and dementia, a continuous and vigorous effort from the scientific community is needed to fill substantial knowledge gaps.
